# Nanoscale Lithography Mediated by Surface Self-Assembly of 16-[3,5-Bis(Mercaptomethyl)phenoxy]hexadecanoic Acid on Au(111) Investigated by Scanning Probe Microscopy

**DOI:** 10.3390/molecules190913010

**Published:** 2014-08-25

**Authors:** Xianglin Zhai, Han Ju Lee, Tian Tian, T. Randall Lee, Jayne C. Garno

**Affiliations:** 1 Department of Chemistry, Louisiana State University, Baton Rouge, LA 70803, USA; 2 Department of Chemistry and the Texas Center for Superconductivity, University of Houston, Houston, TX 77204-5003, USA

**Keywords:** multidentate thiol, self-assembly, nanofabrication, particle lithography, nanofabrication

## Abstract

The solution-phase self-assembly of bidentate 16-[3,5-bis(mercapto-methyl)phenoxy]hexadecanoic acid (BMPHA) on Au(111) was studied using nano-fabrication protocols with scanning probe nanolithography and immersion particle lithography. Molecularly thin films of BMPHA prepared by surface self-assembly have potential application as spatially selective layers in sensor designs. Either monolayer or bilayer films of BMPHA can be formed under ambient conditions, depending on the parameters of concentration and immersion intervals. Experiments with scanning probe-based lithography (nanoshaving and nanografting) were applied to measure the thickness of BMPHA films. The thickness of a monolayer and bilayer film of BMPHA on Au(111) were measured *in situ* with atomic force microscopy using *n-*octadecanethiol as an internal reference. Scanning probe-based nanofabrication provides a way to insert nanopatterns of a reference molecule of known dimensions within a matrix film of unknown thickness to enable a direct comparison of heights and surface morphology. Immersion particle lithography was used to prepare a periodic arrangement of nanoholes within films of BMPHA. The nanoholes could be backfilled by immersion in a SAM solution to produce nanodots of *n*-octadecanethiol surrounded by a film of BMPHA. Test platforms prepared by immersion particle lithography enables control of the dimensions of surface sites to construct supramolecular assemblies.

## 1. Introduction

Considerable effort has been directed toward studies of monothiol-based self-assembled monolayers on gold; however, the surface self-assembly of multidentate thiol adsorbates has received far less attention, particularly at the molecular level. The stability of organosulfur-based adsorbates to oxidation, heat, and exposure to light can limit the durability of surface films [1,2,3,4,5,6,7,8,9,10]. Multidentate SAMs derived from thiol adsorbates exhibit enhanced thermal and chemical stability compared to those derived from *n-*alkanethiols [8,11]. Having additional thiol moieties in the headgroup of these adsorbates enables a chelate effect, which improves the stability of the films [12,13].

**Figure 1:**
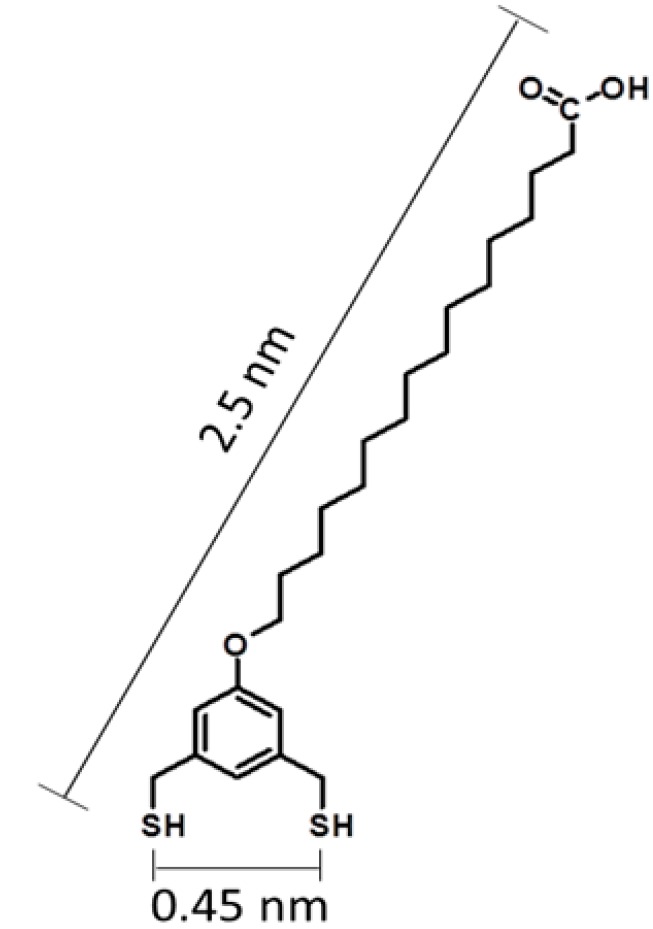
Structure of 16-[3,5-Bis(mercaptomethyl)phenoxy]hexadecanoic acid (BMPHA).

Previously, we investigated the surface self-assembly of tridentate 1,1,1-tris(mercaptomethyl)-heptadecane with *in situ* studies using atomic force microscopy (AFM) [14]. The adsorption of tridentate adsorbates proceeds via a more complex assembly pathway than that of monothiols since the multidentate molecules require successive steps to form S–Au bonds to the surface. The bidentate molecule selected for this study is 16-[3,5-bis(mercaptomethyl)phenoxy]hexadecanoic acid (BMPHA), shown in Figure 1 [15]. The design of BMPHA incorporates two thiol groups placed at *meta-* positions of a phenyl moiety, connected by bridging methylene groups. The distance between the two thiol groups (5 Å) was designed to place the two sulfur atoms at binding sites of Au(111) without substantial torsional strain [1,16]. The molecular backbone of BMPHA consists of a chain of sixteen carbons terminated with carboxylic acid. The acid moiety can be used as a linker moiety for binding proteins [17] or metals [18]. By changing immersion conditions, electrostatic interactions at the interface can produce head-to-head linkages of acid endgroups to form bilayer films [19].

Scanning probe based protocols of nanoshaving and nanografting have been applied to measure the local thickness of organic films [19,20,21], for monitoring adsorption kinetics on surfaces [22,23], to identify functional groups of *n*-alkanethiols [24,25], and to confine spatially the deposition of DNA [26,27], proteins [17,28,29], and metals [18]. Nanofabrication based on scanning-probe methods requires steps of rastering the probe to write each pattern individually. A high-throughput approach for nanolithography with organic thin films has been developed that can generate billions of circular nanopatterns at once, using surface masks of close-packed monodisperse mesospheres. Particle lithography enables the manufacture of billions of regular nanopatterns with well-defined geometries based on solution self-assembly and steps of drying and evaporation [30,31]. Arrays of regularly-shaped nanostructures provide a foundation for further chemical reactions and are useful for local AFM characterizations. For example, the areas that were masked by mesospheres furnish exposed sites of Au(111) for depositing organothiols to generate multicomponent surface patterns with selected functional groups.

The synthesis of multidentate thiol-based adsorbates offers opportunities for generating robust interfaces of well-defined structure and composition. In our experiments, nanostructures of BMPHA were prepared with close packed silica spheres on Au(111). Essentially, our strategy was to construct spatially-selective containers of exposed sites of the substrate to direct the subsequent deposition of a second molecule. Samples prepared by nanolithography with organic thin films enable side-by-side comparisons of the surface structures of multidentate adsorbates *versus* *n*-alkanethiol monolayers (*i.e.*, film thickness, periodicity, surface properties). Surface characterizations were accomplished using *in situ* imaging with AFM to provide fundamental measurements at the molecular level. Scanning probe based lithography experiments employed a liquid sample cell for AFM studies, since fresh reagents can be introduced to the system and step-wise surface changes before and after nanofabrication can be monitored *in situ*. The multidentate SAMs provide a foundation for constructing more complex assemblies at the nanoscale. Side-by-side imaging of the surface structures of BMPHA *versus* *n*-octadecanethiol were accomplished with AFM. A bilayer of BMPHA was formed on Au(111) by changing the immersion conditions (*i.e.*, concentration and immersion time). The thickness of monolayer and bilayer BMPHA thin films on Au(111) was evaluated by measurements with nanoshaving, nanografting and immersion protocols with particle lithography.

## 2. Results and Discussion

Several protocols with scanning probe microscopy were conducted to investigate the self-assembly and surface morphology of BMPHA on Au(111) in a liquid environment. Our goal was to obtain side-by-side views of the morphology of BMPHA and *n*-alkanethiols films using AFM characterizations of grafted nanopatterns. The known height of *n*-octadecanethiol (ODT) was used as a nanoscale ruler to evaluate the thickness of BMPHA films prepared under selected conditions. Depending on the concentration and immersion intervals, either single or double layers of BMPHA could be generated. 

### 2.1. Nanoshaving within a Monolayer Film of BMPHA

**Figure 2:**
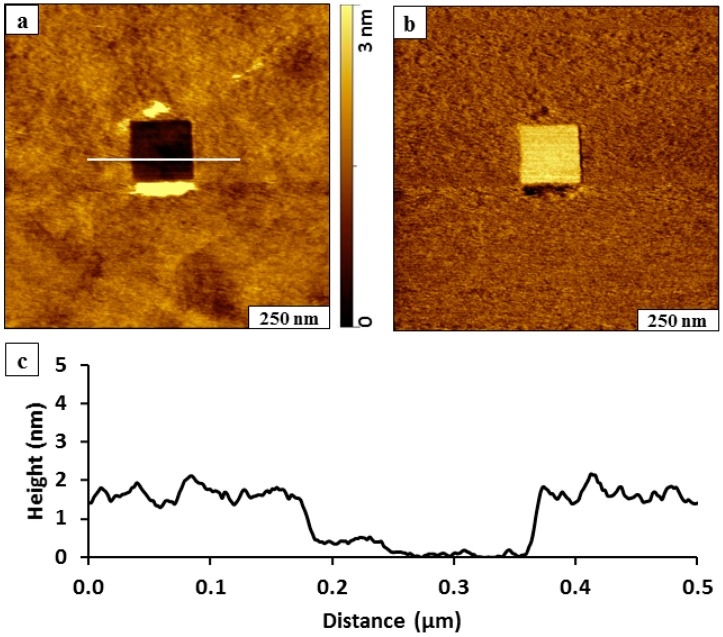
Nanoshaving within a BMPHA monolayer. (**a**) Topography view of nanoshaved square; (**b**) corresponding lateral force image; (**c**) height profile for the white line in ***a***.

A square nanoshaved pattern (200 × 200 nm^2^) within a SAM of BMPHA is shown in Figure 2. The BMPHA film of the matrix area surrounding the nanoshaved hole was formed by 24 h immersion in 0.1 mM solution. The nanoshaved area is the dark square in the topography image (Figure 2a) and reveals the underlying gold substrate. The irregularly shaped bright patches at the top and bottom of the nanoshaved square are residues of BMPHA removed by the AFM probe. To assess how effective the nanoshaving parameters were for removal of BMPHA, the simultaneously acquired lateral force image is shown in Figure 2b. The bright area of the square nanopattern located at the center of the image indicates the exposed areas of the substrate, which indicates clean removal of BMPHA. The tip-surface interactions mapped in lateral force frames are distinct for the gold surface and carboxylic endgroups of BMPHA. The thickness of the BMPHA film can be evaluated with a local cursor measurement, referencing the uncovered area of the substrate as a baseline at the bottom of the nanoshaved square pattern (Figure 2c). The thickness of the BMPHA SAM measured 2.0 ± 0.2 nm, which is in close agreement with the value obtained by ellipsometry [15]. A conservative error term of 0.2 nm is reported due to the roughness of the underlying gold substrate, which is based on the height of a single gold terrace step.

Compared to monodentate *n*-alkanethiols, a higher force was required to shave away the multidentate BMPHA layer. For the example in Figure 1, the area was swept 20 times with 5 nN/m applied force. Typically, *n*-alkanethiols can be nanoshaved with 4-6 sweeps at forces between 0.2 and 10 nN/M depending on the sharpness of the AFM probe. When nanoshaving *n-*alkanethiol SAMs in ethanol, the displaced molecules are often dissolved in the surrounding liquid media so that no residues are present at the edges of the patterns. However, with the bidentate example shown in Figure 2, BMPHA molecules formed aggregate assemblies that failed to dissolve fully in ethanolic media. Intermolecular associations between the carboxylic acid groups and π-π interactions of the aromatic moiety of BMPHA are sufficiently strong to induce aggregation, as indicated by surface residues at the edges of the nanoshaved pattern.

### 2.2. Nanografting n-Alkanethiols within a Monolayer of BMPHA

**Figure 3:**
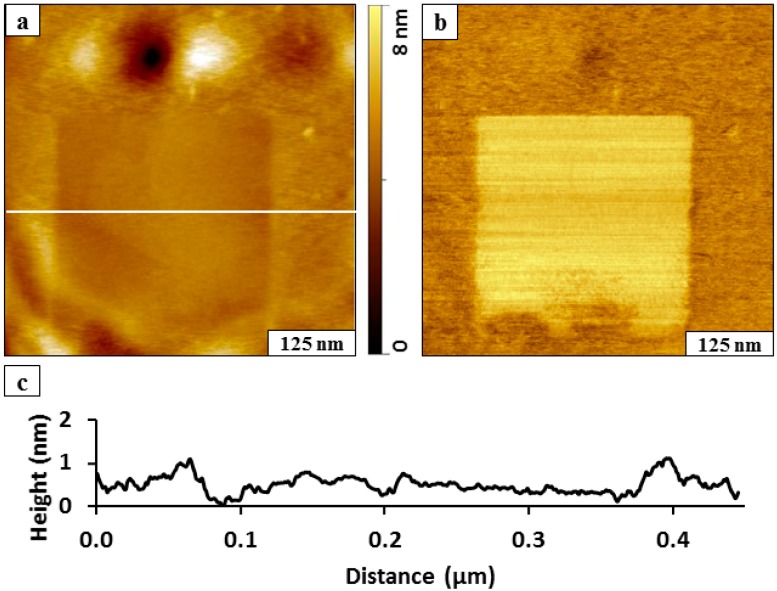
Side-by-side comparison of the surface morphology of ODT and a BMPHA monolayer prepared on Au(111). (**a**) Nanografted pattern of ODT (300 × 300 nm^2^) viewed with a contact-mode AFM topography image acquired in ethanol; (**b**) corresponding lateral force image; (**c**) cursor profile for the line in ***a***.

The thickness and quality of the bidentate BMPHA films were examined further using protocols of nanografting. Films of BMPHA were prepared on Au(111) by immersing a gold substrate into an ethanolic solution (0.1 mM) for 24 h. A nanopattern of *n*-octadecanethiol (ODT) was inscribed within a matrix of BMPHA (Figure 3) to enable a side-by-side comparison of the surface morphology. The nanopattern of ODT appears to be slightly shorter in height than the surrounding areas of BMPHA (Figure 3a) revealing a recessed square region containing overlapping gold steps. The concurrently acquired lateral force image (Figure 3b) reveals distinct changes in the surface chemistry for the regions of the ODT nanopattern and BMPHA matrix. The horizontal lines are produced by the left and right raster pattern of the AFM probe. The expected height for a densely packed ODT monolayer is 2.1 nm, assuming a tilt angle of 28°. Residues of BMPHA are piled at the edges of the nanografted pattern and provide a distinct boundary around the inscribed region of ODT; therefore, comparison of the height differences at the pattern edges cannot be used to provide a reliable estimate of the BMPHA film thickness for this example. Comparing the cursor measurement in areas beyond the edges of the nanopattern reveals that the thickness of the BMPHA film is approximately the same as ODT, which would correspond to a monolayer.

The areas of BMPHA appear to have a rougher morphology in the areas surrounding the nanografted ODT pattern (Figure 3a). A few small adsorbates are present on the BMPHA areas, and the density of BMPHA is consistent with a loosely packed tailgroup assembly [31]. The differences in tip-surface adhesive interactions are quite distinct in the lateral force image of Figure 3b. A monolayer of BMPHA would present carboxylic acid moieties at the surface, whereas the nanopattern of ODT is terminated with methyl groups, which produces distinct changes in color contrast for the lateral force frame.

**Figure 4:**
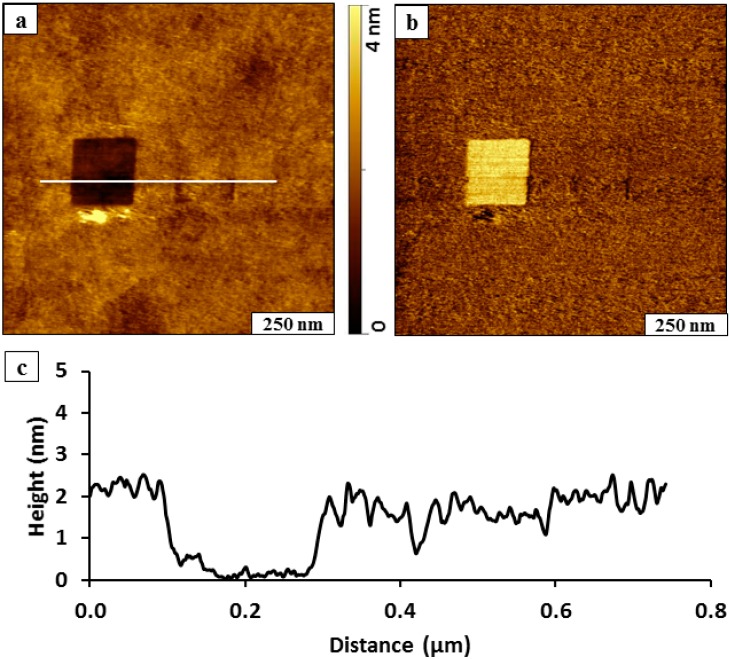
Fabrication of a nanoshaved square and nanografted pattern of BMPHA side-by-side within a monolayer film of BMPHA. (**a**) Contact-mode topography image acquired in ethanol; (**b**) lateral force frame; (**c**) cursor profile for the line in ***a*** drawn across both nanopatterns.

Protocols of nanoshaving and nanografting can be accomplished within the same experiment by rinsing and exchanging solutions with the AFM liquid sample cell. This protocol requires a stable imaging environment, since it is easy to perturb the sample to displace the tip away from the nanofabricated region. In the next *in situ* experiment, a 200 × 200 nm^2^ square of BMPHA molecules was nanografted within a naturally grown BMPHA SAM (Figure 4). The nanopattern can be vaguely distinguished near the center of the image, because the bottom corners of the pattern did not fill in completely. Otherwise, the height of the nanografted area matches the height of BMPHA and is not visible in the topography frame. After nanografting, the BMPHA solution was removed and replaced with clean ethanol to enable nanoshaving. A square area (200 × 200 nm^2^) was swept at high force to disclose the underlying gold substrate at the left side of the image. The nanoshaved area is easy to distinguish as a dark square at the left side of the topography frame of Figure 4a.

Although mostly indistinguishable, the square pattern of BMPHA was nanografted immediately to the right of the nanoshaved pattern. The area in the center of the pattern with a nanografted pattern of BMPHA has the same surface chemistry (‑COOH) as the surrounding matrix and cannot be clearly detected in the lateral force image (Figure 4b). However the nanoshaved square on the left side of the frame is readily identifiable with brighter color. 

Combining protocols of nanografting followed by nanoshaving is particularly useful to test if the nanografting solution interacts to bind to the surface of the matrix layer. A representative line profile across the nanoshaved area of Figure 4a shows that the thickness of the film measures 2.1 ± 0.2 nm, referencing the bottom of the nanoshaved area as a baseline (Figure 4c). This thickness corresponds to the expected height for a monolayer of BMPHA; consequently, there is no evidence of adsorption of the nanografting solution to the BMPHA matrix. For the nanografted area, no difference in height was detected for the nanografted patch of BMPHA *versus* the surrounding BMPHA film. This area can be detected using the vague dark outline of the bottom corners of the nanografted square. Considering the overall theoretical molecular length of BMPHA (2.53 nm); the tilt angle for a BMPHA monolayer would measure ~34 degrees, assuming that both sulfurs of the bidentate molecule bind to the surface of gold.

### 2.3. Nanografting ODT within a Double Layer of BMPHA

**Figure 5:**
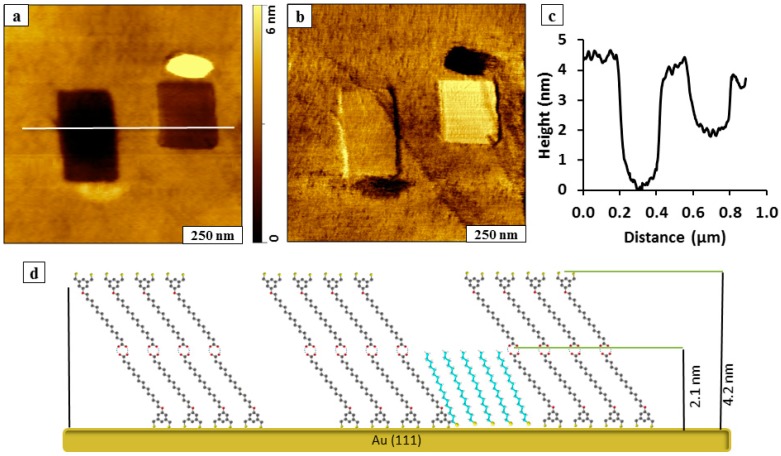
A nanoshaved area and nanografted pattern of ODT placed side-by-side within a BMPHA bilayer. (**a**) Topography image; (**b**) lateral force frame; (**c**) cursor profile for ***a***; (**d**) proposed height model.

A double layer film of BMPHA on Au(111) can be produced by changing the experimental parameters of concentration and immersion intervals. A bilayer of BMPHA was formed by immersion of a gold substrate in 5 mM ethanolic solution for 30 h. Nanopatterns were fabricated within a BMPHA bilayer using sequential steps of nanoshaving and nanografting prepared as shown in Figure 5. First, a rectangular area (200 × 300 nm^2^) was nanoshaved within the BMPHA film. Next, a solution of ODT (1 mM) was injected into the sample cell, and a smaller rectangle was nanografted to the right of the original nanoshaved area. At this concentration, we were unable to detect the growth of ODT within the nanoshaved area over a period of less than one hour. Growth of ODT within nanoshaved regions is greatly influenced by whether the area of the gold substrate is cleanly shaved. The nanoshaved area is not a pristine gold surface for self-assembly and is not well-suited for studying adsorption kinetics. For the rectangular nanopattern on the right side of Figure 5a, an aggregate of residue removed from the nanoshaved area persists near the bottom of the rectangle. The nanografted pattern of ODT has a taller mound of removed adsorbates at the top edge of the feature. Differences in surface adhesion are mapped in the lateral force frame of Figure 5b, revealing different color contrast for the matrix of BMPHA, the nanoshaved area, the nanografted ODT pattern, and adsorbate residues. The patterns exhibit different depths within the BMPHA matrix, as shown with a representative line profile in Figure 5c. The nanofabricated areas have heights that are shorter than the surrounding BMPHA film. The thickness of the BMPHA matrix film measured using the depth of the nanoshaved rectangle is 4.2 ± 0.2 nm, which corresponds to a bilayer. The difference in thickness for the nanografted pattern on the right measured 2.1 ± 0.2 nm, which is consistent with the known thickness for a monolayer of ODT. Additional examples of nanoshaved and nanografted patterns produced within a BMPHA bilayer are provided in supporting information (Figure S1).

A model for the heights of the nanoshaved and nanografted patterns is presented in Figure 5d. In the proposed model, a bilayer is formed by interactions between –COOH functional groups of BMPHA to form a head-to-head arrangement. Previously, double layers were detected with extended immersion or higher concentrations of acid-terminated *n*-alkanethiol SAMs, such as 11-mercaptoundecanoic acid and mercaptohexadecanoic acid [19]. For this model the bilayer of BMPHA presents dithiol moieties at the interface. Notably, no BMPHA bilayer structures were observed when the SAMs were formed from 1 mM ethanolic BMPHA followed by *ex situ* rinsing with water, THF, and ethanol [31].

The optimized threshold force was not determined for each experiment, and depends sensitively on the sharpness of the AFM probe. The amount of force used for fabrication using either nanografting or nanoshaving protocols were comparable for the monolayer or bilayer films of BMPHA. The monolayer film in Figure 3 was fabricated using an applied force of 5.2 nN, whereas the bilayer film shown in Figure 5 was accomplished with a force of 4.4 nN.

### 2.4. Nanofabrication Experiments with BMPHA Using Immersion Particle Lithography

**Figure 6:**
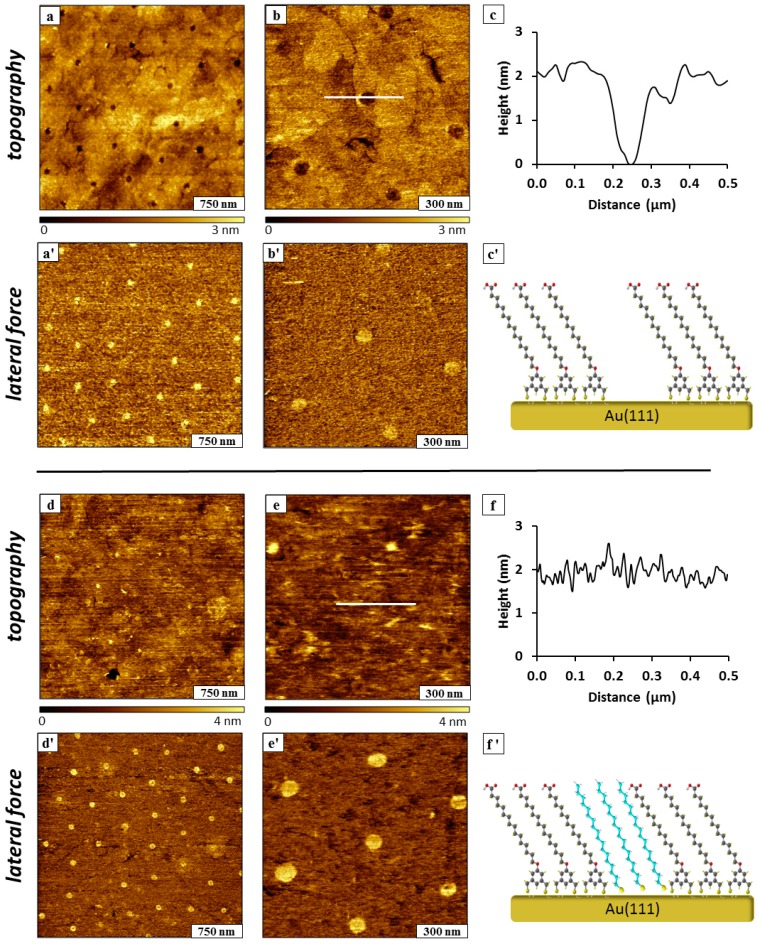
Nanopatterns prepared with ODT and BMPHA using immersion particle lithography. (**a**) Nanoholes within BMPHA; (**a'**) lateral force image; (**b**) successive zoom-in topograph; (**b'**) lateral force image; (**c**) cursor profile for the line shown in ***b***; (**c'**) structural model of BMPHA nanostructures. (**d**) Nanoholes filled with ODT; (**d'**) corresponding lateral force frame; (**e**) zoom-in view of ODT nanopatterns; (**e'**) lateral force image; (**f**) cursor profile for the line in ***e***; (**f'**) chemical model of backfilled ODT within BMPHA.

Using immersion particle lithography, periodic arrangements of nanostructures were produced through surface self-assembly of BMPHA and ODT on gold. Solution self-assembly of thiol-based molecules on gold substrates enables construction of thin films with well-defined dimensions and composition. Particle lithography enables exquisite design of interfacial chemistry by defining the surface coverage of component molecules [30,32]. For protocols with BMPHA, a mask of mesospheres was placed on template-stripped gold (TSG). Two immersion steps were used to prepare islands of ODT within a matrix monolayer of BMPHA. In the first immersion step, the masked TSG substrate was submerged in an ethanolic solution of BMPHA to prepare nanoholes. When the mesosphere mask was removed, a periodic arrangement of uncovered areas of substrate is disclosed (Figure 6a–c). The nanoholes were filled in by a second immersion step with ODT (Figure 6d–f). This protocol produced circular islands of methyl-terminated ODT surrounded by a matrix monolayer of acid-functionalized BMPHA.

Representative AFM images of samples prepared with the two immersion steps are presented in Figure 6; the top panels are views of the nanoholes within BMPHA produced by particle lithography, and the bottom panels were acquired for the same sample after backfilling with ODT. A periodic arrangement of nanoholes is shown in Figure 6a; the dark circles of the topography frame are areas of uncovered substrate and can be more clearly distinguished in the lateral force frame (Figure 6a'). There are approximately 25 nanoholes within the 3 × 3 µm^2^ area, which would scale to an approximate surface density of 10^8^ nanostructures/cm^2^. The spacing between nanopatterns measures 500 nm, matching the diameter of the silica mesospheres used for patterning. With immersion particle lithography, the sizes of the nanoholes are exquisitely small because the areas where the beads make physical contact are much smaller than the periodicity of the mesosphere surface mask. The shape of the nanoholes is more clearly revealed in zoom-in views of Figure 6b,b'. Six sites of nanoholes are shown within the 1.2 × 1.2 µm^2^ frame, defining the areas where the mesospheres were displaced. The topography frames also resolve the shapes of the edges of steps and terraces of the gold substrate beneath the BMPHA film. The depth of the nanoholes measures 2.1 ± 0.2 nm, shown with a representative cursor profile in Figure 6c. A side-view model for a monolayer of BMPHA with areas of uncovered gold is shown in Figure 6c'.

The nanoholes were backfilled with ODT by immersing the sample in a solution of 1 mM ODT in ethanol for 24 h. Changes in surface morphology are readily apparent after backfilling, the locations of the circular holes are no longer visible in the topography frames of Figure 6d,e. Since ODT and BMPHA are similar in height, there is no clear height difference revealed in the topographs, as shown with an example cursor profile in Figure 6f. However, the simultaneously acquired lateral force frames disclose distinct differences in color contrast for the –COOH groups of BMPHA compared to methyl-terminated ODT. Lateral force images enable visualization of the locations and sizes of the ODT nanopatterns in Figure 6d',e'. Using the lateral force frames, the surface coverage of methyl and acid headgroups was determined to be ~5%.

**Figure 7:**
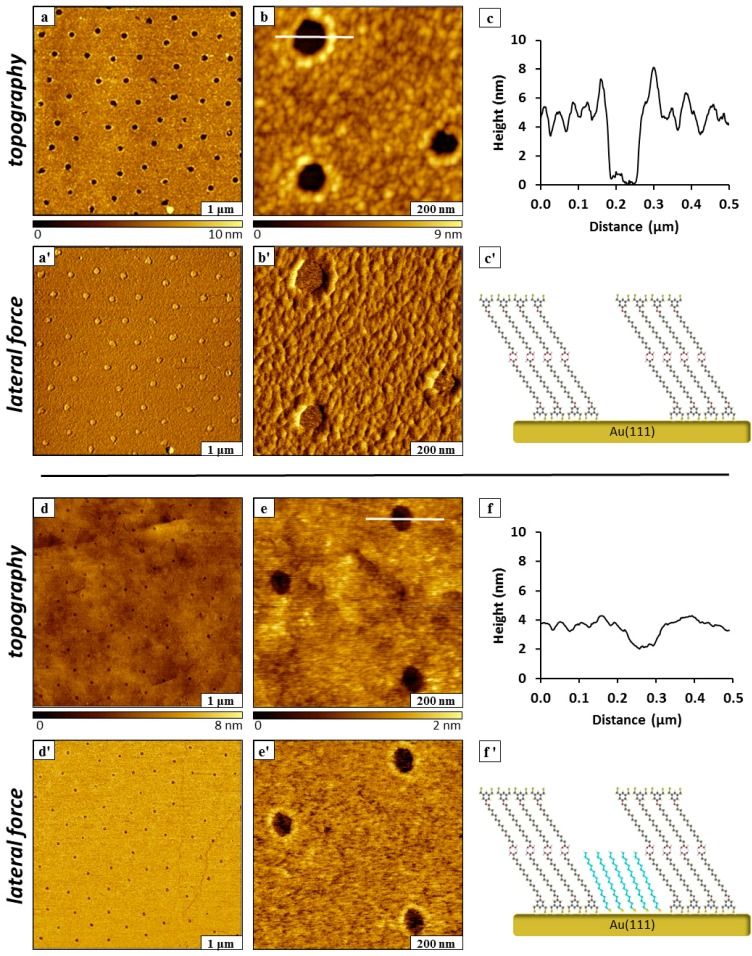
Nanopatterns prepared in a bilayer of BMPHA using immersion particle lithography. (**a**) Nanoholes exposing TSG within a BMPHA bilayer; (**a'**) corresponding lateral force image; (**b**) zoom-in view; (**b'**) lateral force image; (**c**) cursor profile for the line shown in ***b***; (**c'**) chemical model of BMPHA bilayer. (**d**) Nanoholes filled in with ODT; (**d'**) corresponding lateral force frame; (**e**) zoom-in view of ODT backfilled nanopatterns; (**e'**) lateral force image; (**f**) cursor profile for the line in ***e***; (**f'**) chemical model of backfilled ODT within a bilayer of BMPHA.

Immersion particle lithography was similarly applied for making islands of ODT within a bilayer film of BMPHA (Figure 7). The bilayer was produced by using higher concentration of 4 mM BMPHA immersed for 30 h. Approximately 60 nanoholes of uncovered TSG are visible within a BMPHA bilayer for a 5 × 5 µm^2^ area, as shown in Figure 7a,a'. The diameter of the nanoholes measures 110 ± 10 nm, with a surface coverage of 5%. A close-up view of three nanoholes is shown in Figure 7b,b', revealing a somewhat rougher texture at the surface of the BMPHA bilayer. The reference features of the underlying gold substrate cannot be distinguished in Figure 7b as was apparent for the monolayer film of BMPHA (Figure 6a,b). Self-assembly of a monolayer of BMPHA is mediated by binding through S-Au chemisorption to the gold substrate, whereas for the bilayer, the second layer is formed through electrostatic head-to-head interactions between molecules. One might predict that a bilayer film would be less densely packed than a monolayer, and the surface morphology revealed in Figure 7b indicates that the packing density of the BMPHA bilayer has changed in comparison to a monolayer film. The depth of nanoholes formed within the BMPHA was measured to be 5.0 ± 1.2 nm (Figure 7c). This distance is slightly longer than expected for a double layer of BMPHA, possibly attributable to adsorbates on the surface of the bilayer. A model of the side-view of the BMPHA bilayer is presented in Figure 7c', showing head-to-head interactions between carboxylic acid groups that associate to form the double layer structure.

After backfilling the nanoholes with ODT, the patterns still appear as holes within the matrix film of the BMPHA bilayer (Figure 7d,e). Images shown in the lower half of Figure 7 were acquired after 24 h immersion in 1 mM ODT in ethanol. The rougher texture of the BMPHA bilayer appears to be smoother because of additional steps of rinsing and sonication to remove loose adsorbates.

Features of the underlying gold substrate such as step edges can be vaguely resolved for areas of the BMPHA bilayer in the zoom-in view of Figure 7e. Chemical maps of the areas of nanopatterns with methyl headgroups are provided by the lateral force images (Figure 7d',e'). The depth of backfilled nanoholes within BMPHA multilayer was measured to be 1.8 ± 0.2 nm (Figure 7f), which closely matches the expected difference in thickness between a BMPHA bilayer and ODT. The measurement of nanopattern depths extrapolates to a local thickness of 3.9 ± 0.2 nm for a BMPHA bilayer. The proposed model for the height of the double layer pattern is shown in Figure 7f'.

## 3. Experimental Section

### 3.1. Materials

Octadecanethiol was purchased from Sigma Aldrich (St. Louis, MO, USA) and used as received. The new adsorbate of interest, 16-[3,5-bis(mercaptomethyl)phenoxy]hexadecanoic acid (BMPHA) was synthesized as previously reported [15]. Ethanol (200 proof) was obtained from Pharmco-AAper Alcohol and Chemical Co. (Shelbyville, KY, USA) and used as the diluent for preparing thiol solutions. Gold pellets (99.99% purity) were purchased from Ted Pella (Redding, CA, USA).

### 3.2. Preparation of Organic Thin Films on Au(111)

To prepare films of BMPHA for scanning-probe based nanolithography protocols, commercially obtained gold substrates were rinsed with ethanol and submerged in thiol solutions for specifically designated immersion intervals. Films of BMPHA were rinsed with ethanol and then characterized with AFM with protocols of nanoshaving and nanografting. Flame-annealed gold films on mica substrates (150 nm thickness) were purchased from Agilent Technologies (Chandler, AZ, USA).

### 3.3. Preparation of Template-Stripped Au(111)

Template-stripped gold (TSG) films on glass substrates were prepared as reported previously [33]. Gold pellets were deposited in a high-vacuum thermal evaporator (Angstrom Engineering Inc., Kitchener, OR, USA) at 10^−7^ Torr onto freshly cleaved pieces of Ruby muscovite mica (S&J Trading Inc., Glen Oaks, NY, USA). The mica was preheated to 350 °C prior to gold deposition using quartz lamps mounted behind the sample holder. A deposition rates of 3 Å/s was used to prepare films of 150 nm overall thickness. After deposition, the gold substrates were annealed at 365 °C under vacuum for 30 min and cooled to room temperature. The gold film was glued to glass slides as previously reported by Hegner *et al.* [34]. The gold substrates and glass slides were rinsed with deionized water and placed into a UV-ozone generator for 30 min. Epoxy (EPO-TEK, Billerica, MA, USA) was mixed (1:1) and immediately deposited onto the glass slides. The glass slide was placed onto the gold surface so that the drop of epoxy formed a thin film without any air bubbles. The samples were then heated in oven at 150 °C for 2 h to anneal the epoxy. After cooling to room temperature, the glass pieces were carefully peeled from the mica to form TSG.

### 3.4. Immersion Particle Lithography with Masks of Silica Mesoparticles

Size-sorted, monodisperse silica mesospheres (500 nm diameter, Thermo-Fisher Scientific, Waltman, MA, USA) were deposited on TSG as surface masks for immersion particle lithography. Aqueous solutions of silica mesospheres were cleaned by centrifugation to remove surfactants or contaminants. A volume of 300 μL of the silica mesosphere solution was placed into a microcentrifuge tube and centrifuged for 20 min at 20,000 rpm. A solid pellet formed at the bottom of the centrifuge tube, and the supernatant was removed and replaced with deionized water. The pellet was resuspended with deionized water by vortex mixing. The washing cycle was repeated four times. The concentration of silica mesospheres used as surface mask was 2% weight/volume. Freshly-stripped pieces of TSG were cleaned by rinsing copiously with ethanol. A drop (10–15 μL) of the mesosphere solution was deposited onto the TSG substrates and dried under ambient conditions to form surface masks for nanolithography. The mesosphere masks were annealed by heating at 150 °C for 12 h; the heating step enabled the masked substrates to be immersed in solvent solutions for preparing films on TSG. The mesospheres could be removed in a later step by sonication in ethanol. Further details for the procedure of particle lithography with immersion are described with Supplemental Figure S2.

### 3.5. Atomic Force Microscopy

Models 5500 and 5420 scanning probe microscopes (Agilent Technologies) equipped with PicoView v1.12 software were used for AFM characterizations. Images were acquired using contact mode in a liquid cell containing ethanolic solutions. Imaging and nanofabrication were accomplished with silicon nitride tips with an average spring constant of 0.6 N/m (Bruker Instruments, Camarillo, CA, USA). A scan rate of 1 line/s with a typical image force less than 1 nN were used for AFM imaging. A liquid cell made from polycarbonate was used for nanoshaving and nanografting experiments. Digital images were processed and analyzed with Gwyddion v. 2.30 [35].

### 3.6. Scanning Probe-Based Nanolithography (Nanoshaving and Nanografting)

**Figure 8:**
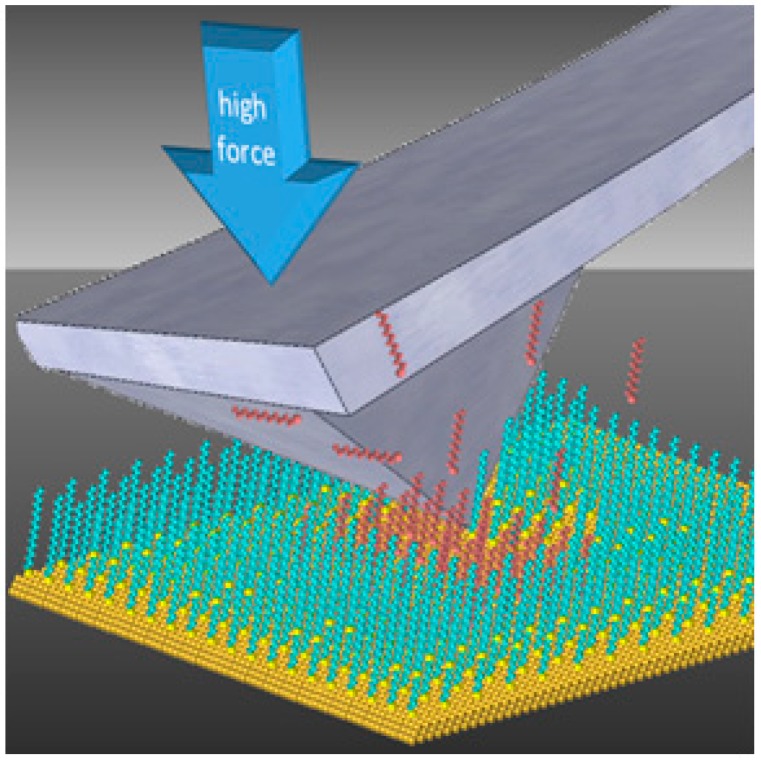
Operation of an AFM probe under force to accomplish nanografting.

Nanoshaving and nanografting experiments with SAMs were done as previously described [36,37]. To accomplish nanoshaving, the probe is swept multiple times across a small region with a higher mechanical force applied to the AFM tip. The sweeping action of the probe is used to remove or displace molecules of the matrix SAM. The area can be characterized *in situ* using the same probe by returning to low force for nondestructive AFM imaging. The amount of force that is required and the number of times needed to cleanly sweep an area needs to be evaluated for each experiment, depending on the sharpness of the probe. Nanoshaving can be done in either ambient air or in a liquid environment. In air, the molecules are displaced to form piles at the edges of the nanoshaved patterns. In liquid media, the shaved molecules can be swept away to produce clean edges at the sides of nanopatterns. Nanografting is accomplished in a liquid environment that contains the selected molecules for patterning. A single sweep of the selected area at high force is used to remove molecules of the matrix SAM simultaneously as molecules from solution self-assemble onto uncovered areas of the substrate. Returning to low force for *in situ* imaging the nanografted patterns can be characterized using the same AFM probe. The fabrication step of nanografting is depicted in Figure 8. Advantages of nanoshaving and nanografting are that a liquid environment enables *in situ* studies of surface changes with high resolution. The nanopatterns that are produced in the fabrication steps can be imaged under low force with the same AFM tip as a tool for imaging and nanofabrication.

## 4. Conclusions

Protocols for AFM-based nanolithography as well as immersion particle lithography were used to evaluate the morphology and thickness of bidentate BMPHA on Au(111). The established thickness of monodentate ODT was used as a reference for *in situ* measurements of film thickness. Either monolayer or bilayer films of BMPHA can be formed on Au(111) by controlling the parameters of solution concentration and the duration of immersion in ethanolic solution. The thickness of monolayer films of BMPHA measured 2.0 ± 0.2 nm, which is consistent with previous measurements reported using ellipsometry. Assuming that both thiols of BMPHA bind to gold, this value would correspond to a tilt angle of 34° from the surface normal. Head-to-head dimerization between interfacial carboxylic groups can produce bilayers of BMPHA, as has previously been demonstrated for *n*-alkanethiols with carboxylic acid groups. Both monolayer and bilayer films of BMPHA could be generated using immersion particle lithography. Periodic island nanopatterns of ODT were grown within nanoholes of BMPHA, constructed with a successive step of sample immersion in SAM solution. Future directions for this research will be to develop protocols to compare the long-term stability of bidentate *versus* monodentate structures using nanofabricated test structures.

## Supplementary Materials

Additional AFM images of nanoshaving and nanografting experiments accomplished within a BMPHA bilayer (Figure S1) and further details for the protocols for immersion particle lithography (Figure S2) are provided as Supporting Information. Supplementary materials can be accessed at: http://www.mdpi.com/1420-3049/19/9/13010/s1.

## Supplementary Files

Supplementary File 1
