# Supplementary material for: Nanoscale Lithography Mediated by Surface Self-Assembly of 16-[3,5-Bis(Mercaptomethyl)phenoxy]hexadecanoic Acid on Au(111) Investigated by Scanning Probe Microscopy
Source: Molecules. 2014 Aug 25;19(9):13010–26. doi: 10.3390/molecules190913010 (PMC6271246; doi:10.3390/molecules190913010)

Supporting Materials

Figure S1. Nanopatterns fabricated by scanning probe based nanolithography within a bilayer of 16-[3,5-bis(mercaptomethyl)phenoxy]hexadecanoic acid (BMPHA). (**a**) Side-by-side views of nanoshaved and nanografted patterns. These figures present a zoom-out view of Figure 5. (**a**) Contact-mode topography image acquired in ethanol. The areas 1, 2 and 3 are nanoshaved regions; areas 4 and 5 are nanografted patterns of n-octadecanethiol (ODT). (**b**) Simultaneously acquired lateral force image; (**c**) cursor profiles corresponding to the red and blue lines in ***a***. The blue line reveals depth of the nanoshaved area, the red line profiles the depth of nanografted pattern. The bilayer of BMPHA was prepared by immersion of a Au(111) substrate in 5 mM ethanolic BMPHA solution for 30 h. The concentration of ethanolic ODT solution used for nanografting was 1 mM.

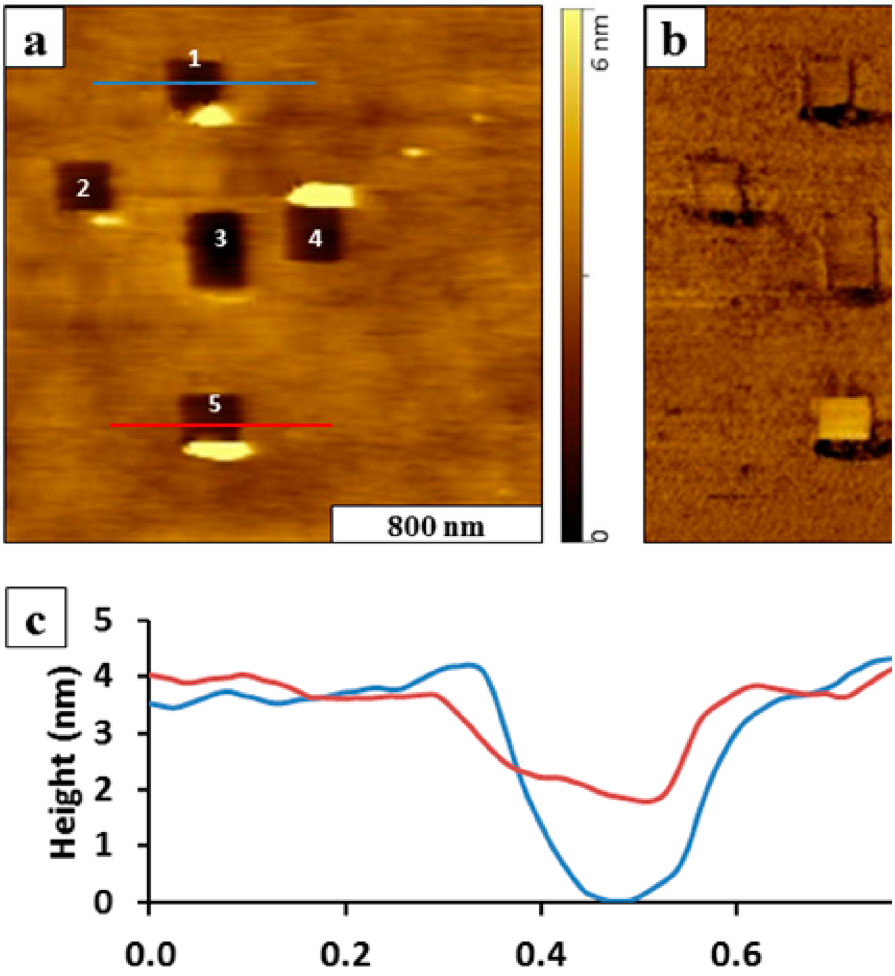

Figure S2. Steps for preparing nanopatterns of thiol-based films using immersion particle lithography. (**a**) A surface mask of close-packed mesospheres was prepared on template-stripped gold, and annealed for 12 h at 150 °C. (**b**) The masked substrate was immersed in an ethanolic solution of dilute multidentate thiol. (**c**) The mask of silica spheres was removed by rinsing and sonication. The uncovered pore areas can be filled with a second molecule, such as n-alkanethiols with a further immersion step.

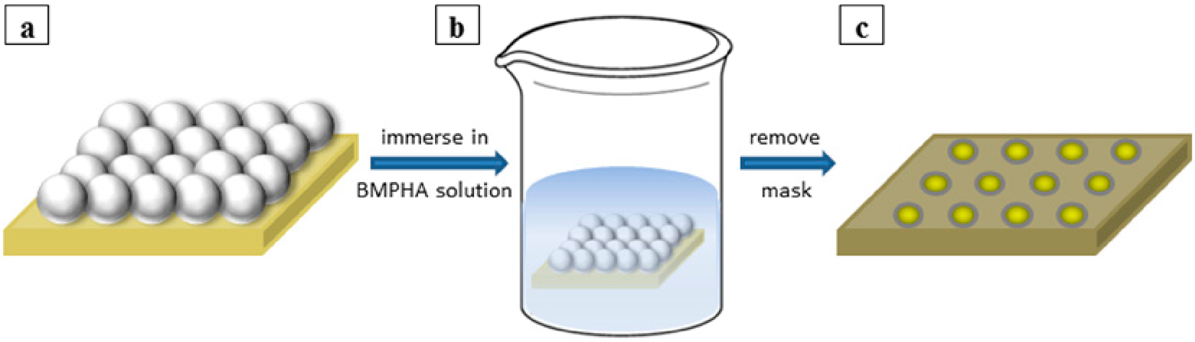

Supplement: Supplementary File 1 [file molecules-19-13010-s001.pdf]
